# Kidney Normothermic Machine Perfusion Can Be Used as a Preservation Technique and a Model of Reperfusion to Deliver Novel Therapies and Assess Inflammation and Immune Activation

**DOI:** 10.3389/fimmu.2022.850271

**Published:** 2022-06-01

**Authors:** Azita Mellati, Letizia Lo Faro, Richard Dumbill, Pommelien Meertens, Kaithlyn Rozenberg, Sadr Shaheed, Corinna Snashall, Hannah McGivern, Rutger Ploeg, James Hunter

**Affiliations:** ^1^ Nuffield Department of Surgical Sciences, University of Oxford, Oxford, United Kingdom; ^2^ Leiden University Medical Centre, Leiden University, Leiden, Netherlands; ^3^ Oxford University Hospital National Health Service (NHS) Foundation Trust, Oxford, United Kingdom; ^4^ University Hospitals of Coventry and Warwickshire National Health Service (NHS) Trust, Coventry, United Kingdom

**Keywords:** normothermic machine perfusion, alpha-1 antitrypsin, inflammatory response, immune activation, cytokines, ischaemia–reperfusion injury, kidney transplantation

## Abstract

Ischaemia–reperfusion injury (IRI) is an inevitable process in transplantation and results in inflammation and immune system activation. Alpha-1 antitrypsin (AAT) has anti-inflammatory properties. Normothermic machine perfusion (NMP) can be used to deliver therapies and may help in assessing the effects of IRI and immunity. This study investigated the effects of AAT on IRI and inflammation in pig kidneys when administered during preservation, followed by normothermic reperfusion (NR) with autologous whole blood, as a surrogate for transplant. Two different models were used to deliver AAT or placebo to paired slaughterhouse pig kidneys: Model 1: 7-h static cold storage (SCS) + 3-h NR (n = 5 pairs), where either AAT (10 mg/ml) or placebo was delivered in the flush following retrieval; Model 2: 4-h SCS + 3-h NMP + 3-h NR (n = 5 pairs), where either AAT or placebo was delivered during NMP. Injury markers and cytokines levels were analysed in the perfusate, and heat shock protein 70 KDa (HSP-70) was analysed in biopsies. AAT delivered to kidneys showed no adverse effects on perfusion parameters. HSP-70 fold changes were significantly lower in the AAT group during NMP (P < 0.01, paired t-test) but not during NR. Interleukin-1 receptor antagonist (IL-1ra) fold changes were significantly higher in the AAT group during NR model 1 (p < 0.05, two-way ANOVA). In contrast to the AAT group, significant upregulation of interleukin-1 beta (IL-1β) and interleukin-6 (IL-6) between t = 90 min and t = 180 min and interleukin-8 (IL-8) between baseline and t = 90 min was observed in the control group in NR model 2 (p < 0.05, Tukey’s multiple comparison test). However, overall inflammatory cytokines and injury markers showed similar levels between groups. Delivery of AAT to pig kidneys was safe without any detrimental effects. NMP and NR provided excellent methods for comparison of inflammation and immune activation in the delivery of a novel therapy.

## Introduction

Chronic kidney disease (CKD) resulting in end-stage renal failure leads to the need for renal replacement therapy (RRT) that includes dialysis or kidney transplantation (1, 2). Kidney transplantation results in improved patient survival and improved quality of life and is more cost-effective (3). However, there is a gap between the availability of organs and the number of patients waiting, resulting in an organ shortage (4). To meet these demands, there is a need to use non-standard organs including those from older donors with comorbidities, so-called expanded criteria donors (ECDs). Donation after circulatory death (DCD) donor kidneys are more prone to delayed graft function (DGF; the need for dialysis after transplant) due to a period of warm ischaemia sustained during retrieval (5). The severity of ischaemia–reperfusion injury (IRI) sustained following transplant in non-standard organs increases the chance of poor long-term outcomes (6–8).

IRI is unavoidable in kidney transplantation, and numerous studies have been involved in understanding mechanisms of IRI using *in vitro* and *in vivo* models (9). IRI consists of pathophysiological conditions including cell death, microvascular dysfunction, transcriptional reprogramming, innate and adaptive immune system activation, and endothelial-to-mesenchymal transition (9, 10). The efficacy of various pharmacological treatments has been tested on renal IRI in several studies. These studies investigate potential protective and improving effects of pharmacological agents on renal IRI through inhibition of different mechanisms including cell death, endothelial cell injury, inflammatory responses, complement activation, and free radical production, as well as supporting mitochondria protection and antioxidant capacities *in vitro*, animal models (*in vivo* and *ex vivo*), and clinical trials (11–17).

While *in vitro* IRI models are restricted by cell type numbers and simulating pathophysiological conditions involved in IRI, small animal species like rodents and rabbits are also limited by size, anatomical differences, and lack of natural heterogeneity due to inbreeding when it comes to translatability to human. Large animal models such as pigs have been used in IRI and transplantation studies due to similarities in genetics, anatomy, and pathophysiology with human as well as their suitability for needed surgical procedures (18). Use of slaughterhouse pigs could be preferable when considering cost and ethics due to the use of non-consumption organs compared to animal house pigs that also have been shown to be a valid DCD model (19).

Normothermic machine perfusion (NMP) is an established preservation method in the research setting, although the mechanisms of action and the impact on inflammation and immunity are unclear. NMP provides near-physiological conditions using oxygenated perfusate at 37°C that creates the opportunity for assessment of organ viability and interventional therapeutics prior to kidney transplantation (20). Alpha-1 antitrypsin (AAT) is a protease inhibitor that is used as an augmentation therapy for patients with AAT deficiency. There is evidence that AAT has anti-inflammatory and tissue protective effects, which may be beneficial in areas such as organ transplant and organ preservation (21–25).

The aim of this study was to investigate the effects of AAT delivered during static cold storage (SCS) or NMP followed by assessment using normothermic reperfusion (NR) on inflammation and immune activation in ischemia–reperfusion in pig kidneys.

## Materials and Methods

### Study Design

Preclinical studies were performed using paired slaughterhouse pig kidneys to simulate the warm ischaemic injury sustained in DCD kidneys. Due to the use of slaughterhouse kidneys in this study, no ethics committee approval was needed.

**Figure 1:**
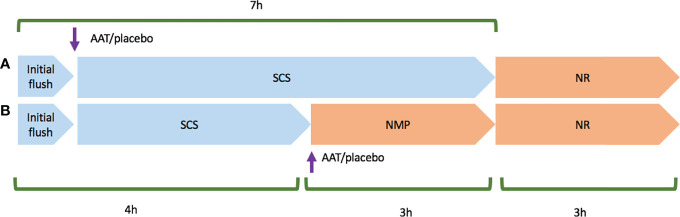
Schematic planning of model 1 and model 2 experiments. Cold ischaemia time started upon the initial flush using Soltran. **(A)** Represents model 1 experiment that Alpha-1 antitrypsin (AAT) or placebo was added in the second flush, and then kidney pairs were stored in static cold storage (SCS) in a mixture of Soltran + Alpha-1 antitrypsin (AAT) or placebo. **(B)** Represents model 2 experiment that following retrieval, kidney pairs were transferred to the lab in cold storage, and then AAT or placebo was added to NMP circuits once both normothermic machine perfusion preservation (NMP) circuits were primed. Both models were followed and completed by 3-h normothermic reperfusion with whole blood (NR).

Two models were designed to deliver AAT or placebo (equivalent human albumin concentration to treatment group) during different preservation methods: Model 1: 7-h SCS + 3-h NR (n = 5); Model 2: 4-h SCS + 3-h NMP + 3-h NR (n = 5). In model 1, following retrieval either the drug or placebo was delivered at the beginning of SCS, and in model 2, the drug or placebo was delivered at the beginning of NMP as the main preservation phase. Both models were completed with 3-h NR as an experimental surrogate for transplantation (**Figure 1**). It was decided that the drug would be given at the beginning of preservation to expose the organ as early as possible. As NMP could not be initiated in the slaughterhouse, the kidneys in model 2 needed a short period of SCS for transport back to the lab and preparation of NMP.

### Retrieval and Surgical Procedures

Model 1 (7-h SCS + 3-h NR): Pairs of porcine kidneys and blood were obtained from a local slaughterhouse. Pigs were slaughtered following Home Office guidance using electrocution and exsanguination *via* jugular vessels. Autologous blood was collected in a container with 20,000 units heparin, and pigs were de-haired in a 60°C hot wash for 5 min. Afterward, both kidneys were flushed with ∼1,000 ml heparinised Soltran (5,000 IU in 1,000-ml Soltran). Here, 4-mm punch biopsies were taken from the upper pole of both kidneys for further analysis. Left and right kidneys were randomly allocated to control or treatment using the randomization function in Microsoft Excel software. A 500-ml solution containing 6.74% AAT (Kamada) and Soltran (final concentration: 10 mg/ml) for treatment and 20% human albumin (Grifols) with 0.9% saline and Soltran (final concentration: 10 mg/ml) for control was produced. Kidneys were flushed with 100 ml of either treatment or control and suspended in the remaining 400 ml of solution. They were then cold stored at <4°C on melting ice for 7 h to simulate the approximate average cold-stored preservation time in clinical practice.

Model 2 (3-h NMP + 3-h NR): Warm allogeneic blood was directly collected into a heparinised funnel (10,000 IU) connected to leucocyte filters to obtain leucocyte depleted blood for the normothermic preservation phase. Kidneys were retrieved as for model 1 and transferred to the lab using SCS. Baseline tissue biopsies were taken immediately prior to the start of NMP.

### Perfusate Preparation for Normothermic Machine Perfusion and Normothermic Reperfusion

#### Normothermic Reperfusion

Whole blood was filtered through medical gauze to remove debris. Filtered blood was diluted to a haematocrit of 30% with 0.9% saline with the following additional supplements: Amoxicillin-clavulanate (1,200 mg) to avoid bacterial contamination, mannitol (10 mg) as a free radical scavenger and osmotic diuretic, insulin (5 IU) to facilitate physiological glucose absorption, and verapamil (0.75 mg) to reduce vasoconstriction. Also, 5% glucose and 10% calcium gluconate were added during NR to maintain physiological values. In this study, 500 ml of perfusate was used for each circuit.

#### Normothermic Machine Perfusion Preservation

A red blood cell (RBC)-based perfusate was prepared using allogeneic leucocyte-depleted blood. Filtered blood was centrifuged at 3,000 × g for 20 min at room temperature (RT), and the plasma supernatant was removed and discarded. To make 1,000 ml perfusate, 750 ml 5% human albumin (Grifols), 9 ml 10% calcium gluconate, 60 ml demineralised water, 18 ml 5% glucose, and 15 ml 8.4% sodium bicarbonate were mixed together, and then isolated RBCs were gradually added to the mixture to reach 25% haematocrit. Afterward, in addition to 500 ml of prepared RBC-based perfusate, amoxicillin-clavulanate (1,200 mg), insulin (5 IU), mannitol (10 mg), creatinine (1,000 µM), and verapamil (0.75 mg) were added to each circuit.

### Perfusion Circuits

The Kidney Assist device (Organ Assist) was used for NMP and NR. This consisted of a membrane oxygenator (Hilte Lt 2500, Medos Medizintechnik or Terumo FX05, Terumo Corp.), a centrifugal pump head (DP3, Medos Medizintechnik AG), and tubing. In this study, 95% O_2_/5% CO_2_ carbogen cylinder (BOC) (0.5 ml/min rate) was used for gas delivery. An ultrasonic flow sensor probe (Em-tec-Gmbh) measured flow rate on the arterial line, and a pressure transducer (Edwards Lifesciences) was connected to the arterial line at the same height as the renal artery. Temperature was set at 37°C and perfusion pressure at 70 mmHg.

Perfusate samples were taken and centrifuged at 1,800 × g for 12 min at 4°C and stored at -80°C freezer. Urine was recirculated during NMP and NR in model 2 but not during NR in model 1. Tissue biopsies were taken at baseline (abattoir for model 1 and before the start of NMP for model 2) and end of NMP and NR. All tissue biopsies were bisected; half was stored in formalin, and half was frozen at -80°C.

### Transferring From Normothermic Machine Perfusion to Normothermic Reperfusion

Once NMP was completed, arterial cannulas were clamped and disconnected from the circuit and kidneys were promptly flushed with Soltran to remove the perfusate. They were then stored cold until the NR phase (temperature <4°C). Simultaneously, NMP circuits were rinsed using 0.9% saline to remove the residual perfusate and primed using NR whole-blood perfusate. This procedure from the end of the NMP phase to the start of the NR phase took ∼60 min.

### Arterial Blood Gas Values and Biochemistry Analyses

Arterial blood gas values were measured using ABL90 FLEX blood gas analyser. Perfusate samples were sent to the clinical biochemistry lab (John Radcliffe Hospital, Oxford, UK) to determine aspartate aminotransferase (AST) and lactate dehydrogenase (LDH) activity.

### Neutrophil Gelatinase-Associated Lipocalin

NR perfusate samples were used to determine the concentration of neutrophil gelatinase-associated lipocalin (NGAL) using a pig Lipocalin-2 ELISA kit (Abcam, Cat. No.: ab207924). In brief, perfusate samples were added in duplicate to the precoated kit wells. Standards and blanks were also added to the plate, and the plate was incubated for 1 h at RT. Afterward, all wells were properly washed using diluted wash buffer and then a biotinylated pig-NGAL primary antibody was added, followed by incubation for 1 h at RT. After washing, horseradish peroxidase (HRP)-streptavidin was added to all wells and the plate was incubated for 1 h at RT on the shaker. 3,3′,5,5′-Tetramethylbenzidine (TMB) and hydrogen peroxide substrates were added to all wells once washing steps were completed, and after 10-min incubation in the dark, the stop solution was added to the plate and the plate was read using a Bio-Rad iMark microplate reader.

### Kidney Injury Molecule-1

Kidney injury molecule-1 (KIM-1) concentration was measured in NMP and NR samples using Porcine KIM-1 ELISA kit (FineTest, Cat. No.: EP0102). Briefly, the precoated ELISA plate was washed twice using wash buffer, and samples and standards were plated and incubated at 37°C for 90 min. Then, the plate was washed twice, and 100-µl primary antibody (1:100 dilution) was added to each well followed by incubation for 60 min at 37°C. After 3 washes, 100-µl HRP-streptavidin conjugate (1:100) was added to each well. Following this, the plate was incubated again at 37°C for 30 min. Next, the plate was washed and 90-µl TMB solution was added to each well, and the plate was again incubated in the dark at 37°C for 25 min. Then, 50 µl of stop solution was added to each well, and the plate was read in Bio-Rad iMark microplate reader at 450 nm.

### Cytokines

Cytokine concentration was measured in NR samples using a Millipore Luminex kit (Merck, Cat. No.: PCYTMAG-23K). Briefly, samples (baseline, t = 90 min, and t = 180 min) were thawed on ice and centrifuged at 10,000 × g for 5 min and then were diluted to 1:2 (model 1) and neat (model 2). Then, 200-μl assay buffer was added to each well, and the plate was left on a shaker for 10 min. After decanting assay buffer, 25 μl of serially diluted standards, quality controls, assay buffer (background), and samples were added to the wells based on the plate layout. Then, 25-μl assay buffer was added to the sample wells, and 25 μl Serum Matrix was added to background, standards, and control wells. Afterward, 25-μl beads were added to each well, and then the plate was incubated on a shaker overnight in the dark at 2°C–8°C. The next day, the plate was washed 3 times and 50-μl detection antibody was added to each well. After 2-h incubation at RT, 50 μl streptavidin-phycoerythrin was added per well, followed by 30-min incubation. The plate was washed 3 times, and 100-μl Drive Fluid was added to each well. The plate was run in Magpix Luminex machine controlled with xPONENT software. All results were analysed using Milliplex Analyst software.

### Heat Shock Protein 70KDa

The expression of heat shock protein 70 KDa (HSP-70) was measured using Western blot technique. In brief, tissue biopsies were homogenised using Radioimmunoprecipitation assay buffer (RIPA buffer) (Merck) in a sonicator, and then the protein concentration was determined by Pierce™ Bicinchoninic acid (BCA) Protein Assay Kit (Thermo Fisher Scientific). Equal amounts of protein from kidney cortical biopsies were loaded on 26 wells of a 4%–12% sodium dodecyl sulfate–polyacrylamide gel (SDS_PAGE gel), and gel electrophoresis was performed at 80 V for 15 min and then 150 V for up to 2 h at RT. Then, separated proteins were transferred to a Polyvinylidene fluoride membrane (PVDF membrane) membrane at 60 V for 3 h at 4°C. After transfer, the membrane was washed in Tris-buffered saline, 0.1% Tween 20 (TBS-T) and blocked in 5% semi-skimmed milk for 60 min on shaker at RT. The membrane was washed using TBS-T and incubated by primary antibodies HSP-70 (Cat. No.: C92F3A-5, Bioquote-stressmarq) and β Actin (Cat. No.: ab8226, Abcam) diluted in 5% milk-TBST overnight at 4°C. Next day, blots were washed with TBST and then were incubated with IRDye 680RD (Cat. No. 926-68070, Li-Cor) under agitation for 1 h at RT. Blots were washed in TBST and rinsed with 1× TBS and then scanned by Odyssey DLx Imager (LI-COR Biosciences). The scanned blots were analysed by ImageJ software and GelAnalyzer software for quantitation.

### Myeloperoxidase Staining

Immunohistochemistry staining of myeloperoxidase-positive (MPO+) cells was performed by staining MPO on paraffin-embedded kidney slices with 4-μm thickness. Antigen retrieval was performed using Epitope Retrieval Solution for 20 min (Bond, AR9961). Then, sections were stained using rabbit anti-human MPO antibody (1:400, Agilent Technologies, Cat. No.: A039829-2). All staining steps were performed using automatic Leica Microsystem machine.

### Statistical Analyses

The area under the curve (AUC) was calculated for continuous variables. Fold changes were calculated by dividing values by baseline values except where baseline values were below detection range to compare control vs. treatment group. Data were assessed for normality and were analysed using paired t-test or 2-way ANOVA to compare control vs. treatment group. Changes over time were also analysed using 2-way ANOVA. Results are reported as mean ± standard deviation (SD) of the mean. In this study, p values <0.05 were considered as statistically significant. Analyses were performed using GraphPad Prism 9.0.

## Results

### Machine Perfusion Characteristics and Haemodynamics

**Figure 2:**
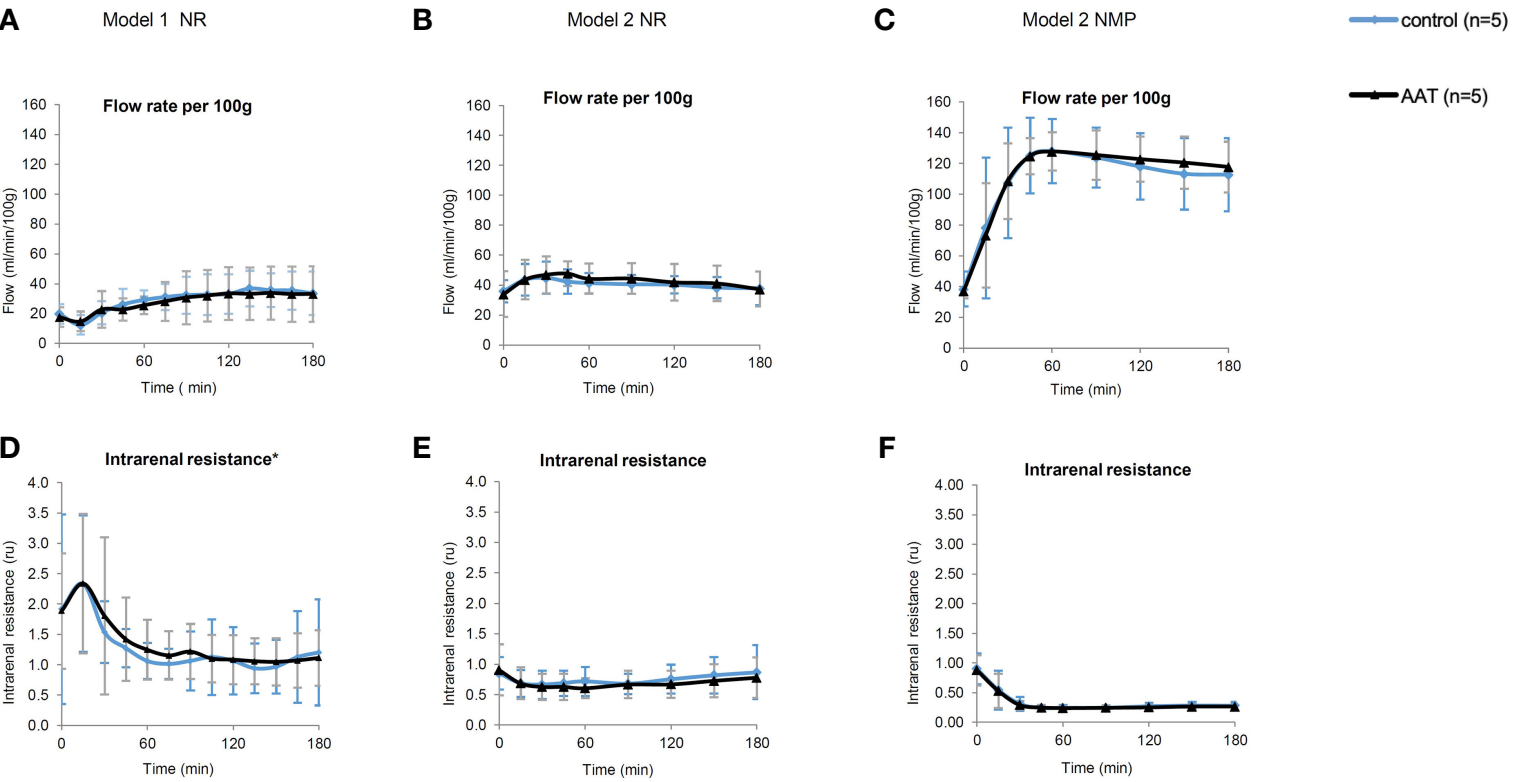
Perfusion parameters including flow rate (ml/min/100 g) and intrarenal resistance (ru). **(A–C)** represent flow rate, and **(D–F)** represent intrarenal resistance during the normothermic reperfusion phase (NR) in models 1 and 2 as well as during the nomothermic machine perfusion preservation (NMP) phase in model 2, respectively. All results are shown as mean ± SD. *One data point (t = 15 min) in the graph **(D)** is excluded due to a technical error in the device.

All kidneys in model 1 and model 2 experiments were perfused successfully and without any adverse events. The mean flow rate decreased during the first 30 min followed by an increase to a steady state in NR phase of model 1. As for model 2 (both NMP and NR), mean flow rate increased from the beginning of perfusion to steady state. Flow rate and intrarenal levels were similar between AAT and control groups in NR (models 1 and 2) and NMP phases (**Figure 2**).

### Kidney Injury Biomarkers

KIM-1 and NGAL that are released following tubular injury in plasma were measured as biomarkers of acute kidney injury (26–28). KIM-1 concentration in plasma increased over time during NR in models 1 and 2 (p < 0.0001 for both models 1 and 2 and p < 0.001 for NMP, 2-way ANOVA) (**Figure S1**) with similar levels in both AAT and control groups. In addition, NGAL levels in plasma increased over time in both models 1 and 2 (p < 0.001 and p < 0.05, respectively, 2-way ANOVA) with similar levels in both AAT and control groups (**Figure S2**). These results suggest that the addition of AAT does not reduce tubular injury following ischaemia–reperfusion. Furthermore, an increase in the levels of both KIM-1 and NGAL during warm perfusion suggests increasing tubular injury during preservation.

AST and LDH are markers of cellular injury, and lactate is a marker of ischaemic injury (28). All 3 increased throughout the 3-h NR (models 1 and 2) and 3-h NMP. The AUC of LDH and lactate levels in model 1 and model 2 (NMP and NR) showed similar levels in the AAT and control groups (**Figure S3**). The AUC of the AST levels during NMP in the treatment and control groups was 144.8 ± 40.9 vs. 179.8 ± 64.5, respectively (p = 0.08, paired t-test).

### Heat Shock Protein 70 KDa

**Figure 3:**
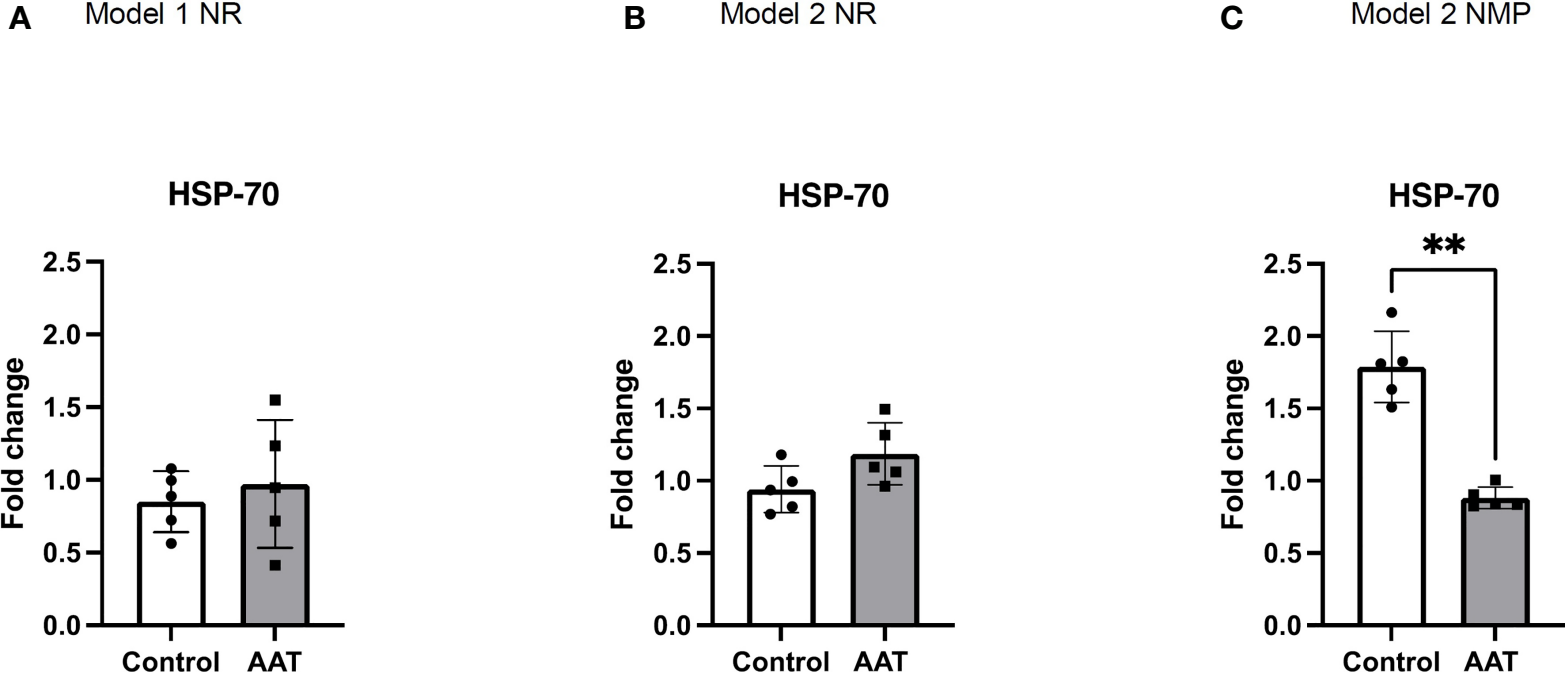
Fold changes of heat shock protein-70 KDa (HSP-70) protein expression in tissue biopsies in normothermic reperfusion (NR) phase following 7-h static cold storage (SCS) in model 1 **(A)** and following 4-h SCS + 3-h normothermic machine perfusion preservation (NMP) in model 2 **(B)** as well as in NMP phase in model 2 **(C)**. Values for HSP-70 were normalised to β actin, and results were normalised to baseline values to calculate fold changes of HSP-70 at the end of NR and NMP. Results are shown as mean ± SD and **p < 0.01.

HSP-70 that increases following ischemia–reperfusion has a direct relationship with elevated levels of tissue injury due to oxidative stress (29–31). Similar levels of HSP-70 were observed in the AAT and control groups in the NR phase of both model 1 and model 2. A significant increase in levels of HSP-70 protein expression was observed in the control group compared to that in the treatment group (p < 0.01, paired t-test), showing less oxidative stress in the AAT group at the end of NMP (**Figure 3**).

### Cytokines

A Luminex assay was used to analyse the inflammatory profile during NR in both model 1 and model 2 experiments.

As for the model 1 experiment, interleukin-1 alpha (IL-1α), interleukin-12 (IL-12), and tumour necrosis factor alpha (TNF-α) significantly decreased over time (p < 0.01 for IL-1α and IL-12, and p < 0.05 for TNF-α, 2-way ANOVA). The levels of interleukin-6 (IL-6), interleukin-8 (IL-8), interleukin-18 (IL-18), interleukin-1 receptor antagonist (IL-1ra), and interleukin-10 (IL-10) significantly increased over time (p < 0.0001 for IL-6, IL-8, and IL-18, and p < 0.01 and p < 0.05 for IL-1ra and IL-10, respectively, 2-way ANOVA). IL-1ra fold changes showed a significant increase in the AAT group compared to those in the control group in model 1 (p < 0.05, 2-way ANOVA). In addition, IL-1ra levels showed a significant increase when levels at t = 90 vs. t = 180 and t = 0 vs. t = 180 were compared in the treatment group in model 1 (p < 0.05, Tukey’s multiple comparison test). This suggests that the addition of AAT during cold storage leads to upregulation of IL-1ra after 90 min during NR. Interleukin-1 beta (IL-1β) and interferon gamma (INF γ) levels stayed steady during NR.

**Figure 4:**
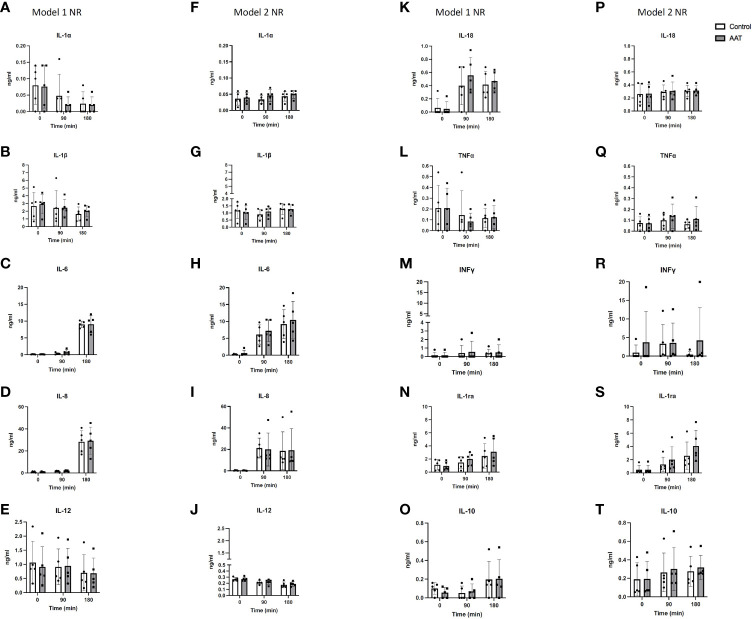
Cytokine concentrations in the perfusate over 3-h normothermic reperfusion (NR) with whole blood following 7-h static cold storage (SCS) in model 1 as well as following 4-h SCS + 3-h normothermic machine perfusion preservation (NMP) in model 2 experiments. Panels **(A–E, K–O)** represent results for model 1 experiments; and panels **(F–J, P–T)** represent results for model 2 experiments. Results are shown as mean ± SD.

In model 2, there was a significant decrease in levels of IL-12 (p < 0.0001, 2-way ANOVA). In contrast, a significant increase in levels of IL-1α, IL-1β, IL-6, IL-8, IL-1ra, and IL-10 was observed over time (p < 0.05 for IL-1α and IL-1β, p < 0.01 for IL-8 and IL-10, p < 0.001 for IL-6 and IL-1ra, 2-way ANOVA). In this model, IL-1β and IL-6 significantly increased between t = 90 min and t = 180 min in the control group (p < 0.01 and p < 0.05, respectively, Tukey’s multiple comparison test). In contrast, in the AAT group, similar significant increases between t = 90 min and t = 180 min were not observed. This suggests that addition of AAT during NMP caused inhibition of IL-1β and IL-6 upregulation in NR after 90 min. In addition, IL-8 levels significantly increased from baseline to t = 90 min in the control group (p < 0.05, Tukey’s multiple comparisons test), while this significant difference between baseline and t=90 min was not observed in the AAT group. This suggests that addition of AAT in NMP inhibited IL-8 upregulation in NR between baseline and t = 90 min. IL-18, TNF-α, and INF γ levels stayed steady without any significant changes. No significant differences were observed between the control and treatment group except for IL-1ra in model 1 (**Figure 4**).

### Myeloperoxidase-Positive Cells Before Preservation and After Normothermic Reperfusion

**Figure 5:**
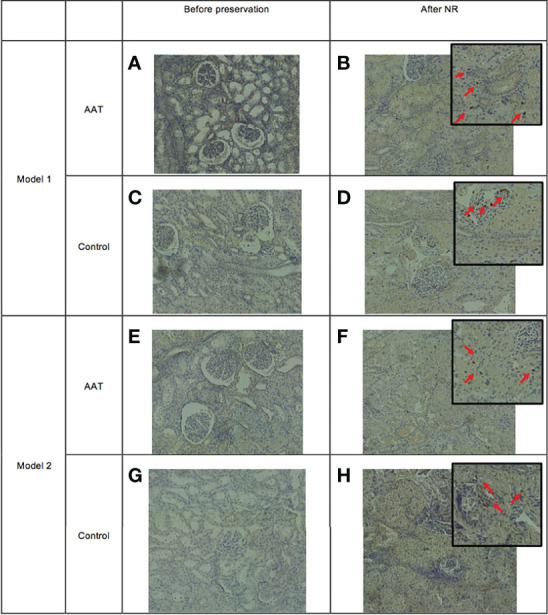
Infiltration of myeloperoxidase-positive (MPO+) cells into tissue following normothermic reperfusion (NR). Panels **(A, C)** represent alpha-1 antitrypsin (AAT) and control groups before static cold storage (SCS), and panels **(B, D)** represent tissue following NR in model 1 (7-h SCS + 3-h NR). Panels **(E, G)** are AAT and control groups before normothermic machine perfusion preservation (NMP), and panels **(F, H)** represent tissue following NR in model 2 (4-h SCS + 3-h NMP + 3-h NR).

Light microscopy of tissue slices showed the number and distribution of stained MPO+ cells present in the tissue in biopsies taken after completion of NR. **Figure 5** shows biopsies taken prior to the start of preservation and biopsies taken after NR. There was an increase in the number of MPO+ neutrophils in the tissue following NR in both models and in both control and treatment groups.

## Discussion

In this study, the efficacy of AAT on IRI has been assessed by delivery of the drug during different preservation methods (NMP and SCS) followed by reperfusion with autologous whole blood. Although NMP has great potential for delivery of the pretransplant treatments, it is much simpler to deliver potential treatments during SCS, and as such, both methods were tested in this series of experiments. Much like other groups, we have used paired kidneys to test the addition of a novel therapy and then used NR, with whole blood as an experimental surrogate for transplant (32–34).

NMP is performed with RBCs suspended in albumin, without leucocytes, complement, platelets, and the immune compartment of the plasma, such as cytokines, chemokines, and other mediators. This allows the technique to be used as a preservation method designed to minimise the influence and injury caused by the immune system during perfusion, although there are still some immune cells present within the graft (35, 36). NR, using whole blood, offers an experimental surrogate to transplant, as the perfusate contains all blood components, and it is possible to be conducted after a long period of cold storage or short normothermic perfusion.

The addition of AAT during two different kidney preservation techniques was safe and feasible. There were indications that AAT might have some early anti-inflammatory and tissue-protective effects with increased levels of IL-1ra and inhibition of IL-1β, IL-6, and IL-8 upregulation during NR. There was also a decrease in AST levels and HSP-70 levels during NMP, which leans toward NMP being a more effective method of delivery for AAT than SCS. However, the majority of cytokines and tissue injury markers assessed were similar in both groups and in both models. The absence of a clear difference in outcomes when AAT was delivered during SCS compared with NMP was surprising. One might expect the normothermic environment to be more favourable to the activity of the enzymatic properties of AAT. However, from a logistical and resource perspective, the delivery of a therapy during SCS is more desirable than the complexities and cost of delivery during NMP. Other groups have demonstrated the merits of drug delivery during SCS (17), and preclinical testing of optimal preservation methods of delivery using NR as a transplant surrogate may be a helpful way of informing future trials.

Roles of pro- and anti-inflammatory cytokines following IRI have been studied widely using *in vivo* models (37–42). IL-1α and IL-1β stimulate an increase of macrophage chemoattractants (37, 41). IL-8 and TNF-α both participate in stimulation of neutrophil tissue infiltration in response to ischaemia–reperfusion (38, 39). This was also observed in our study when following IL-8 upregulation, infiltration of MPO+ cells into tissue was observed. IL-10 and IL-1ra have shown anti-inflammatory and tissue-protective effects by reducing inflammation and apoptosis following IRI (40, 41). In this study, following NR, increasing levels of IL-6, IL-8, IL-10, and IL-1ra and decreasing levels of IL-12 were observed in both models. IL-12 and IL-8 have stimulatory effects on the production of INF γ from T cells and NK cells, and INF γ contributes to Major Histocompatibility Complex (MHC) class I and II upregulation (42). INF γ and IL-12 have been reported to be upregulated in the late phase of IRI, which might explain the decreasing levels of IL-12 and no changes in INF γ levels in the NR phase of both models (42). Interestingly, in a study of leucocyte mobilisation of donor kidney during NMP preservation following 2-h SCS, inflammatory cytokines were upregulated even though a leucocyte-depleted perfusate was used (43). In another study, in the effluent of a high-volume flush following 2-h SCS, INF γ, IL-1β, IL-8, IL-18, and TNF-α were detected (44). These studies in combination with our findings from the NR phase of both models suggest that donor-derived inflammatory events during preservation might aggravate reperfusion injury during transplantation. As such, it is logical to deliver interventional therapeutics with anti-inflammatory and tissue-protective properties in the preservation phase to reduce donor-derived inflammatory events and test their efficacy during NR.

MPO is a lysosomal enzyme and mainly expressed in neutrophils that allows the infiltration of leucocytes to be assessed and is an excellent measure of early innate immune activation during NMP (45). In this study, there was an absence of MPO+ cells following retrieval and prior to preservation in control and treatment groups in both models. Following NR, there was significant tissue infiltration of neutrophils in both control and treatment groups. This is consistent with findings from other studies using NMP and of renal IRI (46–48). Although there was no suggestion that AAT reduced tissue infiltration of neutrophils in this model, it is clear that both NMP and NR are associated with tissue inflammation.

Our models of kidney preservation followed by NR have some limitations. Firstly, the reperfusion phase was only 3 h, while some of the consequences of IRI and changes in inflammatory markers and immunity would occur much later. For example, upregulation of IL-6 in late IRI has anti-inflammatory properties in contrast to its pro-inflammatory role in early IRI (49), or upregulation of INF γ and IL-12 may be observed in the late phase of IRI (42). In IRI studies using *in vivo* models, a significant decrease of injury markers in the AAT group was only observed in late IRI events, and the 3-h reperfusion in this study may have missed these changes (22). In addition, a lack of complete immune system and other organs in this setup limits our insights into IRI events and changes following interventional therapies. Slaughterhouse pig kidneys are from young healthy animals unlike the older comorbid patients who comprise the donor pool, which is a recognised limitation of the model. However, they are an ethical and sustainable resource that offers an excellent experimental model. We acknowledge that the use of a slaughterhouse model presents more variability in comparison to an animal house model, where the environment is more controlled. However, we have refined this method of retrieval over a number of years and previously described the strengths and limitations (50). We have shown acceptable reproducibility in preclinical dose–response studies where kidneys underwent normothermic perfusion as a basis for a clinical trial (17) and have also demonstrated that this method of multiple organ procurement is adequate for comparing different preservation methods in pig pancreases (51). We have not included histological evaluation in this study due to findings from a previous work on slaughterhouse pig kidneys. Experiments of similarly short duration demonstrated global acute tubular injury and were not sufficient to see histological differences between groups.

This study showed that AAT can be effectively delivered during cold storage and NMP to injured pig kidneys. Although some inflammatory cytokine levels were shown to be lower in the AAT-treated kidneys, the majority of cytokines and tissue injury markers were not different and further data are required to determine whether AAT has an important anti-inflammatory effect.

## Data Availability Statement

The original contributions presented in the study are included in the article/**Supplementary Material**. Further inquiries can be directed to the corresponding authors.

## Ethics Statement

Ethical review and approval were not required for the animal study because organs were retrieved from slaughterhouse animals after death (which occurred following standard Home Office procedures), and therefore no additional ethics were required.

## Author Contributions

Research design: AM, LLF, RP, JH. Performance of research: AM, RD, PM, KR, SS, CS, HM. Drafting of article: AM. Critical revision: All authors. All authors contributed to the article and approved the submitted version.

## Funding

This research was funded by Kamada Pharmaceuticals that also supplied alpha-1 antitrypsin for use in this project.

## Conflict of Interest

The authors declare that the research was conducted in the absence of any commercial or financial relationships that could be construed as a potential conflict of interest.

## Publisher’s Note

All claims expressed in this article are solely those of the authors and do not necessarily represent those of their affiliated organizations, or those of the publisher, the editors and the reviewers. Any product that may be evaluated in this article, or claim that may be made by its manufacturer, is not guaranteed or endorsed by the publisher.

## Supplementary Material

Supplementary Figure 1 Kidney injury molecule-1 (KIM-1) measured in plasma and perfusate in NR model 1 **(A, B)**, NR model 2 **(C, D)** and NMP model 2 **(E)**. A, C and E show KIM-1 concentration, and B and E show KIM-1 fold changes at time points=90 min and 180 min when values were normalised to baseline values.

Supplementary Figure 2 Neutrophil gelatinase-associated lipocalin (NGAL) measured in plasma and perfusate samples in NR model 1 **(A, B)** and NR model 2 **(C, D)**. **(A, C)** show NGAL concentration, and **(B, D)** show NGAL fold changes at time points= 90 min and 180 min when all final values were normalised to baseline values. Data were analysed using paired t-test and a P value<0.05 was considered as statistically significant. Results are shown as mean ± SD and each data point shows result from each kidney.

Supplementary Figure 3 Area under the curve of aspartate aminotransferase (AST) and lactate dehydrogenase (LDH) activity as well as Lactate concentration over 3h NR in model 1 **(A, D, G)** and model 2 **(B, E, H)** as well as 3h NMP in model 2 **(C, F, I)**. Data were analysed using paired t-test and a P value<0.05 was considered as statistically significant. Results are shown as mean ± SD and each data point shows result from each kidney.
