# Supplementary figures and images for: Kidney Normothermic Machine Perfusion Can Be Used as a Preservation Technique and a Model of Reperfusion to Deliver Novel Therapies and Assess Inflammation and Immune Activation
Source: Front Immunol. 2022 Jun 1;13:850271. doi: 10.3389/fimmu.2022.850271 (PMC9198253; doi:10.3389/fimmu.2022.850271)

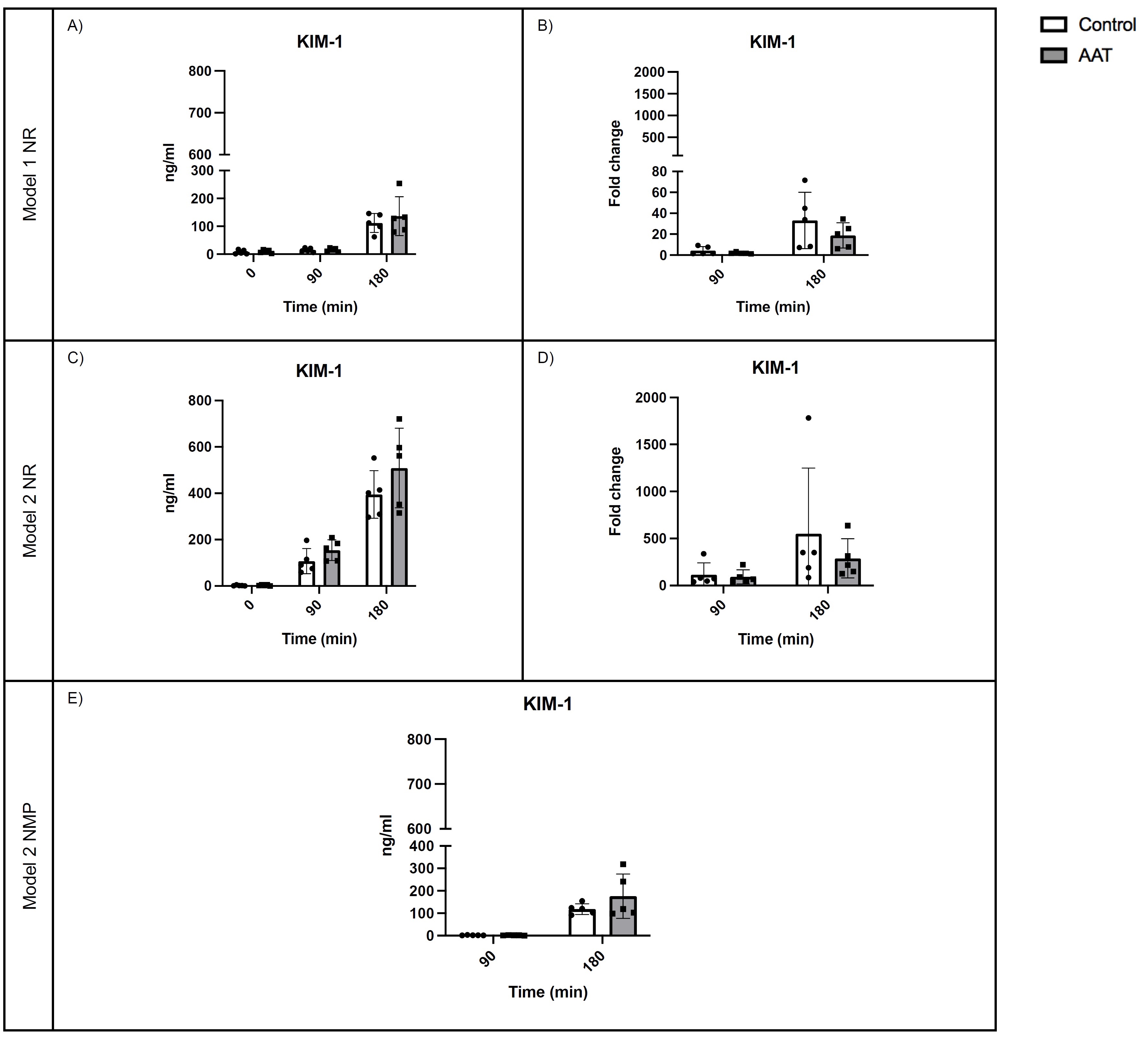

Supplement: Supplementary Figure 1 — Kidney injury molecule-1 (KIM-1) measured in plasma and perfusate in NR model 1 (A, B), NR model 2 (C, D) and NMP model 2 (E). A, C and E show KIM-1 concentration, and B and E show KIM-1 fold changes at time points=90 min and 180 min when values were normalised to baseline values. [file Image_1.jpg]

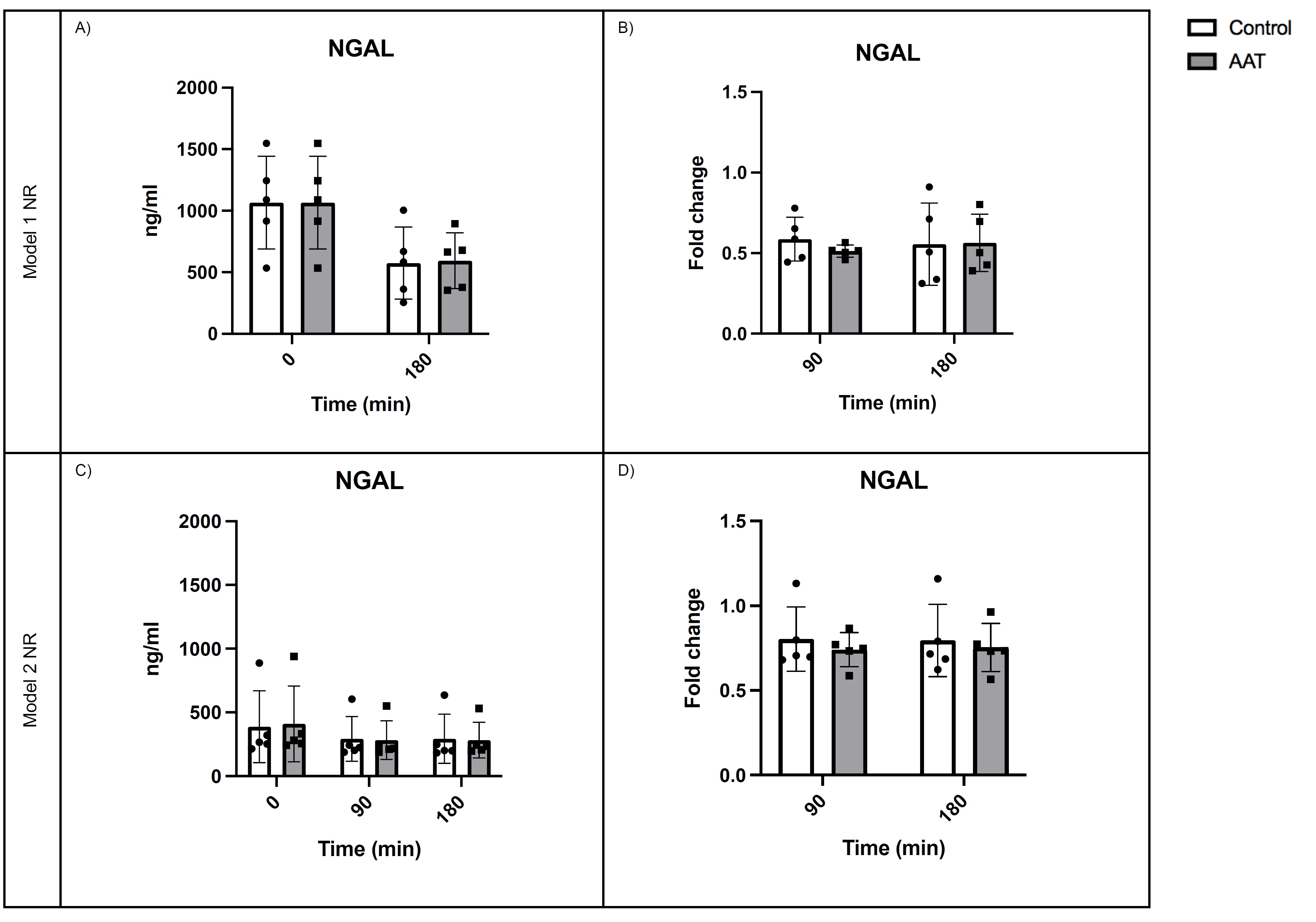

Supplement: Supplementary Figure 2 — Neutrophil gelatinase-associated lipocalin (NGAL) measured in plasma and perfusate samples in NR model 1 (A, B) and NR model 2 (C, D). (A, C) show NGAL concentration, and (B, D) show NGAL fold changes at time points= 90 min and 180 min when all final values were normalised to baseline values. Data were analysed using paired t-test and a P value<0.05 was considered as statistically significant. Results are shown as mean ± SD and each data point shows result from each kidney. [file Image_2.jpg]

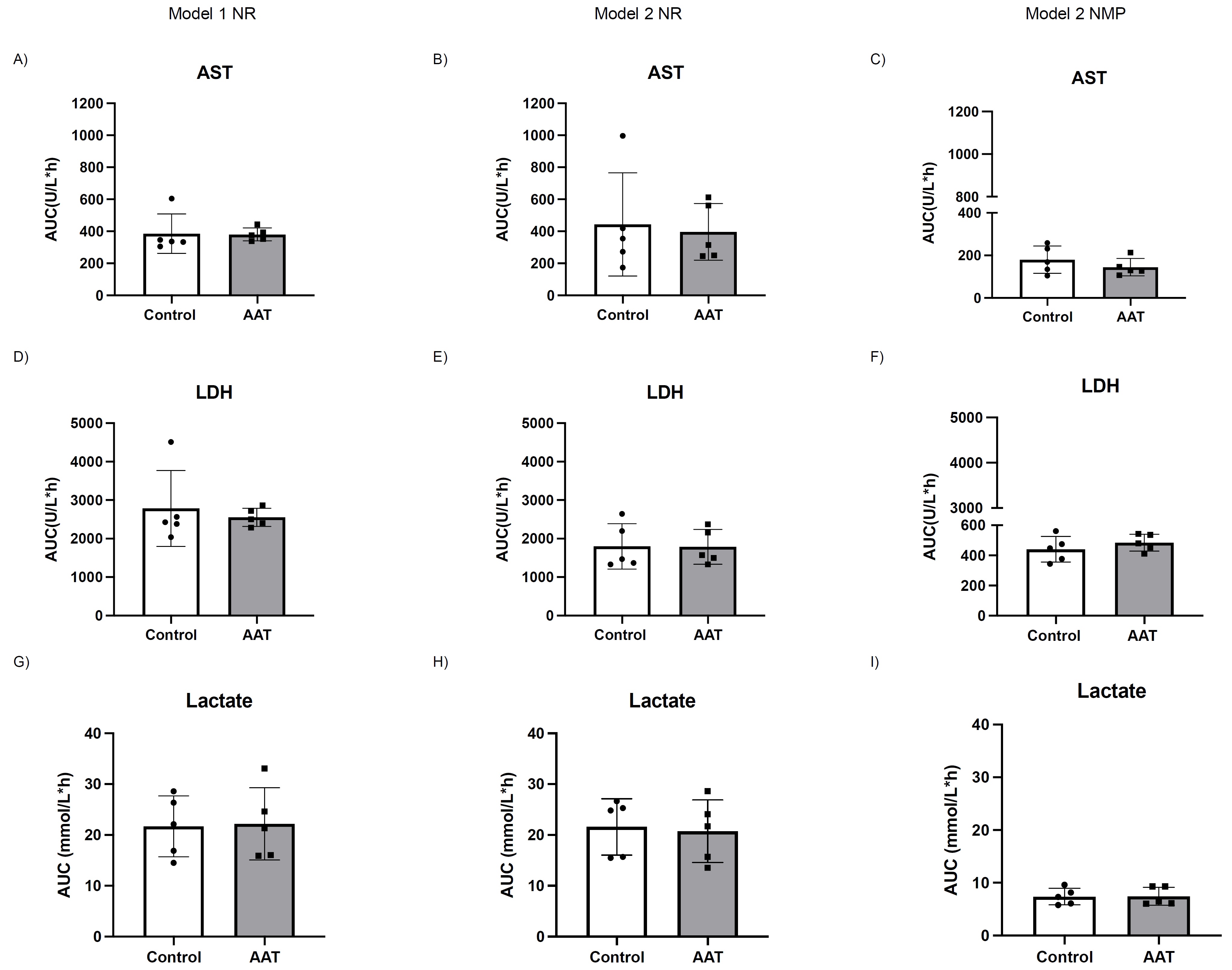

Supplement: Supplementary Figure 3 — Area under the curve of aspartate aminotransferase (AST) and lactate dehydrogenase (LDH) activity as well as Lactate concentration over 3h NR in model 1 (A, D, G) and model 2 (B, E, H) as well as 3h NMP in model 2 (C, F, I). Data were analysed using paired t-test and a P value<0.05 was considered as statistically significant. Results are shown as mean ± SD and each data point shows result from each kidney. [file Image_3.jpg]
